# Rapid urban malaria appraisal (RUMA) in sub-Saharan Africa

**DOI:** 10.1186/1475-2875-4-40

**Published:** 2005-09-09

**Authors:** Shr-Jie Wang, Christian Lengeler, Thomas A Smith, Penelope Vounatsou, Guéladio Cissé, Diadie A Diallo, Martin Akogbeto, Deo Mtasiwa, Awash Teklehaimanot, Marcel Tanner

**Affiliations:** 1 Swiss Tropical Institute (STI), P.O. Box, CH-4002 Basel, Switzerland; 2 Centre Suisse de Recherches Scientifiques (CSRS), 01 B.P. 1303 Abidjan, 01 Côte d'Ivoire; 3 Centre National de Recherche et de Formation sur le Paludisme, (CNRFP) 01 B.P. 2208, Ouagadougou 01, Burkina Faso; 4 Centre de Recherche Entomologique de Cotonou (CREC), Ministère de la Santé Publique, B. P. 06-2604, Cotonou, Benin; 5 Regional/City Medical Office of Health, P.O. Box 9084, Dar es Salaam, Tanzania; 6 The Earth Institute at Columbia University, 215 West 125th St Suite 301, New York NY, 10027, USA

## Abstract

**Background:**

The rapid urban malaria appraisal (RUMA) methodology aims to provide a cost-effective tool to conduct rapid assessments of the malaria situation in urban sub-Saharan Africa and to improve the understanding of urban malaria epidemiology.

**Methods:**

This work was done in Yopougon municipality (Abidjan), Cotonou, Dar es Salaam and Ouagadougou. The study design consists of six components: 1) a literature review, 2) the collection of available health statistics, 3) a risk mapping, 4) school parasitaemia surveys, 5) health facility-based surveys and 6) a brief description of the health care system. These formed the basis of a multi-country evaluation of RUMA's feasibility, consistency and usefulness.

**Results:**

A substantial amount of literature (including unpublished theses and statistics) was found at each site, providing a good overview of the malaria situation. School and health facility-based surveys provided an overview of local endemicity and the overall malaria burden in different city areas. This helped to identify important problems for in-depth assessment, especially the extent to which malaria is over-diagnosed in health facilities. Mapping health facilities and breeding sites allowed the visualization of the complex interplay between population characteristics, health services and malaria risk. However, the latter task was very time-consuming and required special expertise. RUMA is inexpensive, costing around 8,500–13,000 USD for a six to ten-week period.

**Conclusion:**

RUMA was successfully implemented in four urban areas with different endemicity and proved to be a cost-effective first approach to study the features of urban malaria and provide an evidence basis for planning control measures.

## Background

Urbanization has a significant impact on the economy, lifestyles, ecosystems and disease patterns, including malaria [1,2]. An estimated 39% of the population in sub-Saharan Africa (SSA) lived in urban areas in 2003 [3], 198 million Africans lived in urban malaria-endemic areas and 24–103 million clinical attacks occur annually in those areas [4]. An important message addressed in the Pretoria Statement on urban malaria was that the malaria control strategies used in rural areas cannot be directly transferred to the urban context [5]. The epidemiology of urban malaria poses a number of specific challenges: i) the first malaria infection occurs often late in childhood and the acquisition of semi-immunity is delayed [6]; ii) the intensity of the malaria risk is often heterogeneous over small distances, being subjected to the degree of urbanization of particular subdivisions [7,8] and their proximity to possible vector breeding sites [9,10]; iii) rural-urban migration is likely to increase the endemicity of malaria [11]; iv) agricultural and animal husbandry are important economic activities which create a favourable environment for *Anopheles *breeding [12,13]; v) marginalized populations usually lack access to health care, which hampers the effectiveness of case management and the promotion of intermittent antimalarials during pregnancy [5,14-16]. There is now substantial private sector activity in health care provision in many cities. The private services providers are often untrained or unlicensed, but are seen as a source of inexpensive care by patients. There is not much information about the impact of the private sector on case management.

Around 235 papers related to malaria epidemiology in SSA urban settings were published from 1945 to 2004. Entomological profiles and clinical patterns are known to vary between urban, suburban and rural environments [17]. A review of other studies in SSA urban centres showed that transmission patterns vary greatly by city, season and age group. The overall prevalence of parasitaemia was 4.0% in schoolchildren in Brazzaville [18], 2.4–10.3% in Lusaka [19], 2.0% in a Gambian urban area [20] and 3.6–7.5% in Dakar [21]. It was also reported that malaria prevalence in school children varied from 3.0% to 26.4% in different areas of Ouagadougou [22] and varied from 14% in a central urban area to 65% in peri-urban areas in Kinshasa [23].

Evidence showed that the rate of clinical malaria attacks detected in urban health facilities was high and season-dependent. For example, Hendrickse *et al. *found that 36.8% of outpatients were parasitaemic in a hospital in Ibadan [24]. In Niamey, the parasite prevalence was 61.9% during the rainy season but only 5.4% in the dry season in 1989 [25]. In Kinshasa, malaria admissions comprised 29.5% of consultations in 1983, then 38.2% in 1985–86 [26]. In Dakar, malaria fever represented 19.7% of consultations and 34.3% of fever cases were caused by malaria in 1988 [27]; the same authors found that 5.3% (dry season) and 58.8% (rainy season) of febrile outpatients were parasitaemic in 1994 [28]. In Ouagadougou, malaria prevalence accounted for 33% of all outpatients [29], while Dabire reported 22% malaria parasitaemia among children aged 0–14 years in the paediatric ward [30].

Transmission and severity of malaria are influenced by the geographic characteristics of a town and by the socio-economic environment. The heterogeneity and seasonal variation of the entomological inoculation rate, depending on both vector densities and sporozoite rates, have been documented [31,32]. Lindsay *et al. *(1990) showed a difference in the composition of vector species and the vector's adaptation in different subdivisions Banjul [20]. To improve interventions, the determinants of the diversity of transmission levels within subdivisions of a city should be understood. Concerns were raised about the association between urban agricultural activities or local irrigation systems and the creation of breeding sites for *Anopheles *sp. [12,33,34]. Peri-urban areas often lack infrastructure, including poor water supply and sanitation, which provides an ideal environment for vector breeding [35]. For example, urban Dakar has >5,000 market-garden wells which provide permanent sites for mosquito larvae [13]. An identification of vector species, regular larval inspection and larviciding activities should be implemented in the framework of urban malaria control programmes [36].

This article presents the experience of developing a rapid urban malaria appraisal (RUMA) in SSA, carried out with the support of the Roll Back Malaria Partnership. The aims were i) to develop a rapid assessment package that is explicitly evidence-based and can be carried out within a six to ten weeks timeframe; and ii) to assess how rapid malaria appraisal efforts could be best integrated into the municipal health department supervision and to inform control programmes.

## Methods

### Study sites

**Figure 1:**
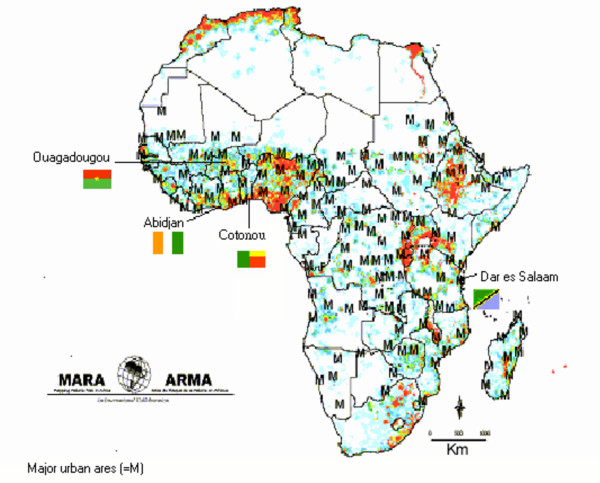
Map of major urban areas in sub-Sahara Africa and the four selected project sites. Major cities (=M) and population density (red >=200, green > 100 and blue = 40 population per square kilometres. Copyright: MARA/ARMA.

The fieldwork took place in Yopougon municipality/Abidjan (Côte d'Ivoire), Ouagadougou (Burkina Faso), Cotonou (Benin) and Dar es Salaam (United Republic of Tanzania) (Figure 1).

Abidjan is the economic capital of Côte d'Ivoire. It is located between latitude 3.7° N–4.0° N and longitude 5.7° E–6.0° E, with a surface area of 454 sq. km. The study was carried out in the large commune of Yopougon (population: 775,000 in 1998) located in the west of Abidjan [37]. The fieldwork in Yopougon municipality (Abidjan) took place from August to September 2002.

Ouagadougou, the capital of Burkina Faso, is situated on the Sahelian border between latitude 12.0° N–13.0° N and longitude 1.15° E–1.40° E. The total surface area was estimated to be around 570–655 sq. km in the year 2000 [38]. The population of Ouagadougou was around 1,100,000 inhabitants in 2002. The fieldwork in Ouagadougou took place from November to December 2002.

Cotonou is the economic capital of Benin. It is located on a strip of land between Lake Nokou and the Gulf of Guinea (between latitude 6.2° N–6.3° N and longitude 2.2° E–2.3° E). The total population was estimated at 780,000 inhabitants on a territory of 73.8 sq. km in 2002 [39]. The fieldwork in Cotonou took place from February to March, 2003.

Dar es Salaam is situated between latitude 6.0° S–7.5° S and longitude 39.0° E–39.6° E on the East African coast. There are 2,500,000 inhabitants on a total surface area of 1,393 sq. km [40]. The fieldwork in Dar es Salaam took place from June to August, 2003.

### Study design

In July 2002, a generic RUMA protocol was developed based on existing urban malaria research protocols [41,42]. The relevant institutions in each setting were contacted and city-specific proposals were then produced. Parts of health facilities mapping, school and health facility-based survey activities were integrated into the routine surveillance and health system evaluation at the municipal level. All the fieldwork was completed in August 2003. Final reports were completed in June, 2004.

The six key components of the RUMA were the following (see also Table 1):

**Table 1:** Study design and methodology of RUMA.

**Key measures**	**Epidemiological measures**			**Spatial relationships**			**Individual variations**			**Institutional factors**		
Methodology	Age-specific morbidity and mortality rates	Fraction of malaria-attributable fevers	Overall endemicity	Gradient of malaria risk	Environmental risks	Travelling history	Socio-economic factors	Bednet usage	Treatment strategy	Public/private partnership	Coverage of treatment providers	Degree of drug resistance

1. Literature review	**x**		x	x	x		x	x	x			x
2. Collection of health statistics	x		x					x	x			x
3. Risk mapping				x	x						x	
4. School parasitaemia survey			x	x	x	x	x	x	x			
5. Health facility-based fever survey	x	x		x	x	x	x	x	x			
6. Brief description of the health care system										x	x	x

1. Literature review. A search of the PUBMED bibliographic database was conducted for the time period from 1960 to April 2004, using the terms "malaria", "urban" and "sub-Saharan Africa". The search was limited to the articles published in English, Chinese, French and Spanish. The reference list of all identified papers was screened. Thesis abstracts filed in the medical libraries of universities and national hospitals were collected at each site and local researchers were also contacted.

2. Collection of routine health statistics. Local experts in ministries of health (MOH) (disease surveillance systems, municipal health departments and national malaria control programmes) and national census and statistics bureaus were contacted to collect demographic data, health system information and statistics, including routine malaria morbidity and mortality reports.

3. Mapping of health care facilities and major *Anopheles *breeding sites. Three or four trained workers carried out the health facility mapping under the guidance of local health personnel. In order to identify *Anopheles *breeding sites, simple larvae sampling was performed with the assistance of entomological technicians in Dar es Salaam and Ouagadougou. The duration of these tasks varied by site: 12 weeks during the rainy season in Dar es Salaam and around three weeks during the dry season in Ouagadougou. Due to security issues and technical problems, the mapping of breeding sites and health facilities could not be performed in Yopougon municipality (Abidjan) and Cotonou.

4. School parasitaemia surveys. School surveys were aimed at determining the local endemicity and risk gradient of malaria. In each city, three to four schools with different malaria endemicity (centre/low, intermediate/medium and periphery/high) were investigated. It is a rapid assessment with limited budget; therefore, in each area only one health facility and school were selected for the surveys. The schools were selected near the selected clinics. 200 school children aged 6–10 years were recruited in each school. Additional information on children was collected using a questionnaire with the assistance of teachers (see Additional file 1).

5. Health facility-based surveys (See Additional file 2). The facility-based fever surveys focused on the age-specific fraction of malaria-attributable fevers [43]. Each city was categorised into three to four areas (centre, intermediate, periphery and rural areas) and one clinic from each area was chosen. Health facilities with a high enough volume of outpatients per day were considered for the survey. In urban areas, an estimated 5% to 50% of fever cases among children under 15 years old were due to malaria. A sample size of 200 in each facility gave an estimate of the proportion of cases with parasites with the following approximate lower 95% confidence limits (at 5%, lower 95% CI: 2; at 50%, lower 95% CI: 6). In each clinic, 200 fever cases and 200 non-fever controls were recruited, with half of them being aged <5 years. Outpatients with a history of fever (past 36 hours) or a measured temperature ≥ 37.5°C were defined as cases. Controls were recruited from another department of the same clinic without current or recent past fever, matched by age and residency.

Electronic thermometers were used to measure the armpit temperature. A "normal" body temperature is referred to as an oral temperature of 37°C. An armpit temperature reading is usually 0.3°C to 0.6°C lower than an oral temperature reading. Therefore 0.5°C was added to the temperature displayed on the digital readout. Thick and thin blood films were taken to identify malaria infections. Using 100× magnification to read the thick smears, all malaria trophozoites and gametocytes were counted separately. Parasite density was calculated according to parasites per 200 white blood cells in a thick film (assuming 8000 white blood cells per ml of blood). If 200 white blood cells were counted and less than 9 malarial parasites found, the counting continued until 500 white blood cells were identified.

6. Brief description of the health care system. It focused on i) the municipal malaria control and prevention efforts, ii) the levels and coverage of service delivery, iii) disease surveillance systems, iv) malaria case management and v) trends of parasite resistance to antimalarials.

### Quality assurance for blood slides

The diagnostic performance and the quality of blood sample readings were checked twice: first in the field and then at the reference laboratory of the Swiss Tropical Institute (STI) in Basel, Switzerland. The results in Yopougon municipality (Abidjan), Dar es Salaam and Ouagadougou were: sensitivity 87.9%, 83.5% and 98.7%; specificity 89.2%, 99.0% and 98.2%; accuracy rate of slide readings 88.8%, 98.5% and 98.6%. The quality control process was not implemented in Cotonou due to operational problems.

### Costing

The financial cost of the resources required for a RUMA were calculated for each site based on local market prices and salary standards, except for the laboratory material that was purchased in Switzerland. All expenses fell into seven categories: salaries, transportation, communications, stationery, laboratory materials, other cost and administrative fees (Table 2). A project team was assembled within the existing structure of partner institutions and then the accountants in each site used a setting-specific cost model to identify the cost factors and determine their local value. The preparation and training cost, programme and administrative costs with the partner institution were estimated and an allowance was added for unforeseen circumstances in the finalized budget. The cost for resources like microscopes and drugs for treatment, vehicles and computers were calculated according to the cost structure of the host institution.

**Table 2:** Budget categories.

**Type of cost**	**Categories**	**Valuation**	**Information source **
Human resources	Project staff Health sector staff	Gross salary Per diem	Salary slips or personnel records from the project office
Transportation	Project vehicles, petrol and maintenance Taxi, motorbike and bus Shipping and packaging	Petrol and maintenance of vehicles based on vehicle logbook Actual expenditure Freight cost	Bills and receipts Tickets and receipts Invoices
Communication	Postage and telephone bills		Bills or contract documents
Stationery	Office maintenance cost Survey materials Photocopies Lap top and printer use	Actual expenditure for items	Agreement with site Agreement and receipts Standard local cost Agreement with site
Laboratory materials & drugs for treatment		International trade good price	Invoices
Other items			Bills and receipts
Administration	Rent of project office, computer and vehicles		Agreement with site

## Results

One of the principal aims of the present work was to review the feasibility, perceived usefulness and consistency of the collected information. Because RUMA was a cross-sectional assessment the external validity of the findings could not be assessed. However, the internal consistency of the results was assessed.

Below, the strengths and weaknesses of each methodology are presented, bearing in mind the constraints imposed by a rapid assessment. Detailed results for each site will be provided in a series of forthcoming publications.

### Literature review (Tables 1 and 3)

**Table 3:** RUMA methodology strengths and weaknesses.

**RUMA Methodology**	**Strengths**	**Weaknesses**
Literature review	• Time-saving, can be done before and afterwards • Can identify qualified local expertise • Comparison of the malaria patterns and trends	• Incomplete information in time and space

Collection of health statistics	• Good description of malaria burden over a longer time period	• Completeness and quality of data

Cross-sectional mapping of healthcare facilities & major *Anopheles *breeding sites	• Visualization of information for policy makers • Helps to plan urban health programmes and upgrade community infrastructure	• Time consuming and only limited scale possible • Breeding sites may be transient /seasonal

School parasitaemia surveys	• Good estimates of local endemicity and local risk factors • Good description of fever prevalence in school • Malaria risk gradient	• Limited representativeness if only small number of schools were sampled

Health facility-based fever surveys	• Estimates malaria-attributable fevers and prevalence of clinical malaria • Description of fever management	• Limited representativeness due to attendance bias

Brief description of the health care system	• Understanding of the structure of city health department and of current malaria control activities • Limited cost • Review of the efficacy of case management	• Only focuses on the available information • Depends on the efficiency of information dissemination within municipal departments

The systematic review of all literature in each city allowed the collection of background information in a time-efficient manner. A substantial body of information was found in each setting, although it was often incomplete in place (for example covering only a part of the city), in time (few time points, only one season) and in content (not all subject areas covered). For the period 1945 to 2004, a total of 109 papers was found (18, 23, 29 and 39 for Abidjan, coastal Benin, Dar es Salaam and Ouagadougou, respectively), relating to malaria epidemiology, socio-economic risk factors of malaria, entomology and drug resistance [44].

### Collection of health statistics (Tables 1, 3 and 4)

**Table 4:** Reported simple malaria cases among total consultations in 4 African cities, all ages. CHU = Centre Hospitalier Universitaire.

**a) Abidjan 2001**									
Communes	Adjamé & Attécoubé	Cocody	Yopougon	Abobo	Plateau	Treichville & Marcory	Port-Bouét & Koumassi	Total	% of admission^†^

Health centers	35,714	55,500	-	71,437	-	-	62,607	225,258	
CHU	No CHU	2,525	-	No CHU	No CHU	12,375	No CHU	14,900	
Total	35,714	58,025	-	71,437	-	12,375	62,607	240,158	40.2
**b) Ouagadougou 2001**									

Sanitary District	Kossodo		Paul VI		Pissy		Secteur 30	Total	% of admission

Total	16,007		24,527		95,868		67,064	203,466	29.3–41.4
**c) Dar es Salaam 2000**									

District hospitals^‡^	Ilala			Kinondoni			Temeke	Total	% of admission

Total	178,016			498,991			395,566	1,072,573	45.4–53.7^‡^
**d) Cotonou 2002**									

Sanitary District	I	II	III	IV	V	VI		Total	% of admission

Total	6,759	9,678	17,339	7,108	29,890	29,483		100,257	32.1–35.9

^†^Reported number of malaria cases divided by the total number of consultations.

^‡^Both Ilala and Temeke district hospitals have malaria reported weekly and monthly. The raw dataset of malaria reports of district hospitals in Kinondoni was missing in 2001. Total numbers of consultations were estimated.

The routine weekly or monthly malaria reports provided a baseline on the burden of malaria in public health facilities, as well as an assessment of the scale of malaria treatment. Overall, case detection in the antenatal clinics and public health services was poor and reporting was not systematic and consistent.

In Abidjan, data were collected from the national malaria control programme (Table 4a). Age-specific monthly data were available. The statistics for 2001 from four out of 10 communes were missing. The malaria cases reported from the main hospitals (Centre Hospitalier Universitaire-CHU) in Yopougon, CHU Cocody and CHU Treichville were separated from the commune data. CHU receive many referral patients and the malaria cases may therefore be over-reported. The data from CHU Yopougon were missing for 2001.

In Ouagadougou, the number of malaria-specific cases and the total number of consultations were collected. The raw data were available by season for 1999–2001, but not for 2002. All the data were missing for Paul VI sanitary district from October to December 2001. The reporting of clinical malaria was also inconsistent in Paul VI (Table 4b).

In Dar es Salaam, the weekly malaria reports were collected from the Ilala, Kinondoni and Temeke district health departments. The data were available for 2000-mid 2003, two months before the survey. A discrepancy in records in Kinondoni District was found, as not all health facilities sent their weekly reports to the district municipal office. Moreover, the sums of reported malaria cases in the raw dataset and in the final district reports were not identical. The Kinondoni district health department had lost all of its 2001 weekly reports (Table 4c).

Only Cotonou had complete data sets for 1996–2002, but the raw datasets were unavailable. Hence, it was impossible to review the consistency and accuracy of the data (Table 4d).

Overall, considerable gaps were found in the routine surveillance systems, particularly for remote health services. Often, the data were collected and presented in different formats, making a generalization impossible and this limited their usefulness. Furthermore, the municipal health departments simply summed up the total numbers of reported cases as they lacked the capacity to analyse these data and to extract useful information for management purposes.

### Mapping activities (Tables 1 and 3)

As stated above, the mapping activities were only done in Ouagadougou and Dar es Salaam.

#### a) Public and private health facilities

In Dar es Salaam, the list of existing public and private health facilities was updated and their locations were recorded by a geographic positioning system (GPS). In Ouagadougou, the mapping of health facilities and schools was done in 2002 by the Ecole Inter-Etats d'Ingénieurs de l'Equipement Rural (EIER), Burkina Faso. Both digital city maps were updated and available for public use.

#### b) Anopheles breeding sites

The malaria risks in Dar es Salaam and Ouagadougou were displayed in relation to the location of health facilities and schools. The mapping of *Anopheles *breeding sites in Dar es Salaam was done on a city wide-scale in conjunction with another project [36,45]. In Ouagadougou, in the limited time available, the focus was on permanent and semi-permanent breeding sites instead of searching for the numerous temporary breeding sites. The produced maps of breeding sites indicated mosquito productivity and distribution in the city in a given season.

The major drawback of mapping is that ground-truthing is very time-consuming and variable over time. During the rainy season, the city-wide larvae collection, larvae hatching and management of data are difficult tasks. Another disadvantage of this approach is that it tends to be very expensive, unless local Geographic Information Systems (GIS) mapping expertise and/or digital city maps are already available for public use. For future studies, it is recommended focusing on the mapping of health facilities and dropping the breeding sites work as it is difficult to assemble a team with the required expertise within such a short time period.

### School parasitaemia surveys (Tables 1 and 3)

**Figure 2:**
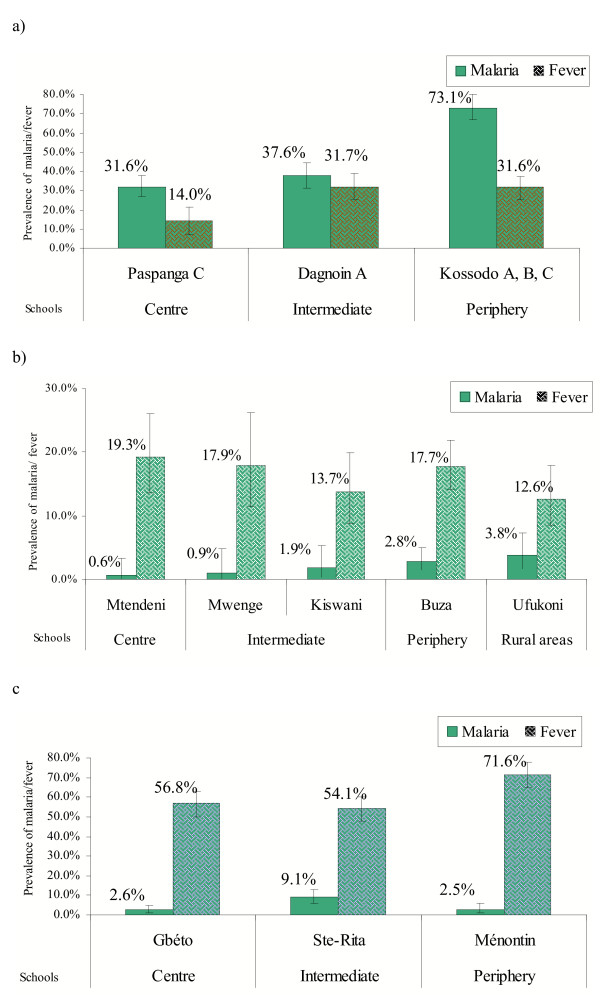
Prevalences of parasitaemia and fever detected in schools, in three sites. The vertical bars represent the 95% CI. a) Ouagadougou. b) Dar es Salaam. c) Cotonou

It was possible to determine the transmission intensity and gradients in different communities. At each site, parasitaemia and fever prevalence rates were obtained for different schools (Figures 2a, 2b, 2c) and by residential areas of children. Around 10 to 70% of children (from city centre to periphery) attended schools with elevated temperature. Malaria prevalence was always higher than the fever prevalence in Ouagadougou since there were many asymptomatic infections. Different communities in Ouagadougou may be exposed to different patterns of malaria transmission and hence the age at first infection and infection patterns may vary. Certainly, the more exposed areas of Ouagadougou experience hyperendemic (if seasonal) malaria. The association between malaria infections and various risk factors were measured and these results are reported elsewhere [46-49].

### Health facility-based surveys (Tables 1, 3 and 5)

**Table 5:** Age-specific malaria prevalence rates in cases and controls by each site. Health facility-based surveys.

Study sites	Abidjan		Cotonou		Dar es Salaam		Ouagadougou	
Age groups/malaria	Cases %	Control %	Cases %	Control %	Cases %	Control %	Cases %	Control %
Infants <1 year	18/78 (23.1%)	22/169 (13.0%)	0/63 (0%)	2/140 (1.4%)	2/99 (2.0%)	4/116 (3.4%)	7/58 (12.1%)	3/21 (14.3%)
Children 1–5 years	61/142 (43.0%)	16/60 (26.7%)	5/68 (6.8%)	4/137 (2.8%)	15/213 (7.0%)	8/178 (4.5%)	45/174 (25.9%)	15/104 (14.4%)
Children 6–15 years	39/89 (43.8%)	8/35 (22.9%)	0/35 (0%)	1/78 (1.3%)	7/97 (7.2%)	2/56 (3.6%)	23/62 (37.1%)	20/58 (34.5%)
Adults >15 years	31/120 (25.6%)	17/119 (14.3%)	2/213 (0.9%)	11/529 (2.0%)	13/308 (4.2%)	8/423 (1.9%)	48/266 (18.0%)	72/363 (19.8%)

Both the fever and control groups (non-febrile admission) had a medium level of parasitaemia prevalence in the health facilities in Yopougon municipality (Abidjan) and Ouagadougou (Table 5). Some people in the control groups reported self-medication with paracetamol or traditional herbs before visiting the clinics. This could have led some malaria cases to present without fever at the clinic. The overall prevalence of malaria was surprising low in Cotonou and Dar es Salaam. This might have been due to high Insecticide Treated Nets (ITNs) coverage and/or the dry climate at the time of survey [46-49].

The detection of malaria parasites in a febrile case does not necessarily indicate clinical malaria. In an effort to improve the case definition and clinical diagnosis, the method of Smith *et al*. [43] was used to estimate the probabilities that individual episodes were really due to a malaria infection. The odds ratio (OR) is the proportion of odds of having parasitaemia in fever cases over controls. The formula for the fraction of fever episodes attributable to malaria parasites is: (1-1/Odds Ratio)*P. P is the proportion of fever episodes in which the subjects had parasitaemia. These age-specific malaria attributable fractions were very low: 0.12–0.27, 0–0.04, 0–0.02 and 0–0.13 in Yopougon municipality (Abidjan), Benin, Dar es Salaam and Ouagadougou, respectively. These results indicated substantial over-treatment at all sites [46-49].

The questionnaires (available as a separate file) administered to cases and controls were tailored for local use. They contained four sections: personal information, economic situation of the family, travelling history, clinical signs and malaria history. The information on age, sex, measured axillary temperature, length of febrile illness, types of previous treatment and the reasons for seeking care were obtained. Stay outside the urban area during the previous three months, the type of housing, urban agriculture activities and ITNs usage were also investigated. These data provided indications of disease perception, preventive measures and socio-economic background at community level.

The questionnaires administered to cases and controls in health facilities were similar to the ones used in school surveys. In all settings the two sets of data were comparable, which allowed for an internal consistency check. For example, in Dar es Salaam 43.1% and 40.2% of households reported ITN use in both the health facility surveys and the school parasitaemia surveys. In Cotonou, these figures were 36.6% and 28.4%, in Ouagadougou 7.8% and 11.1%. The similarity of both surveys also made possible a combined planning and implementation strategy. Detailed results are presented elsewhere [46-49], as well as in a series of forthcoming publications.

### Brief description of the health care system (Tables 1 and 3)

The administrative structures of the national and municipal health departments were sketched out and the list of health facilities was updated at each site. The total numbers of registered malaria diagnosis or treatment providers were: 1060 in Abidjan, 365 in Cotonou, 1684 in Dar es Salaam and 315 in Ouagadougou. Non-governmental organizations and religious hospitals play an important role in health care delivery in Cotonou and Ouagadougou. The catchment areas of all public and private health facilities were further calculated [46-49]. The city malaria control programmes and WHO offices provided information about current malaria control efforts. In order to assess treatment efficacy, the trend of the susceptibility of *P. falciparum *to different antimalarials was reviewed at each site [46-49].

This component required few resources and brought strong political commitment because it involved representatives of the Ministry of Health and the Directors of the municipal health department. The extra-budgetary resources from RUMA helped the local governments to better monitor the provision of health care services, which facilitated an effective exchange of information. The health information was updated but the quality of health care delivery was not assessed because of restricted scope and time. The disadvantage of this approach is that effective communication and dissemination of official documents depends on the attitude of senior officers.

### Compared costing of RUMA activities

The cost for conducting a RUMA in a SSA city with a population of 0.5–3 million is around 8,500–13,000 USD for a six to ten-week period (Table 6). The cost of human resources in Dar es Salaam and Ouagadougou was highest, mainly because of the additional fieldwork performed there (mapping of breeding sites and health facilities). Indeed, the per diem standard was lower in these cities. The higher savings on transportation, communications and materials in Abidjan and Ouagadougou were made possible by our affiliation with local research institutions. The total expense in Abidjan was much lower because the school survey was not performed (the children did not attend school during a politically troubled time). In Cotonou, the excess of human resource and transportation cost was due to unforeseen supervisory expenses.

**Table 6:** RUMA expenses by study sites. QA = Quality control, USD = US dollars 1USD = 650 Francs CFA (Communauté Française Africaine), 1 USD = 1,050 Tanzanian Schilling in 2003.

Sites by the order of total expenses	Human resources	Transport	Communication	Stationery	Lab. materials & drugs	Others	Admin.	Total expenses USD	Total with QA*
Cotonou^§^	2,375	2,942	500	447	793	0	1,000	8,582	No QA
Dar es Salaam	4,321	2,040	93	775	1,030	236	0	8,495	12,435
Ouagadougou	2,493	1,613	198	886	721	98	400	6,970	6,970^¥^
Abidjan	958	411	360	569	707	47	1,000	4,577	7,237

§ without GIS mapping and 1st quality control without GIS mapping and school survey

* with second quality control at Swiss Tropical Institute.

^¥ ^the second quality control was free

In general, the difference in the cost of human resources and communications was due to differences in personnel capacity and fluctuations in the amount of work. The costs of stationery and laboratory materials were less variable, because the needs were the same at each site.

## Discussion and conclusion

This assessment was accomplished in four countries within a period of six to ten weeks in the field and has proven to be a helpful tool in supporting planning of urban malaria control. An ongoing urban malaria control intervention in Dar es Salaam has been initiated on the knowledge basis provided by RUMA. With the incentive of extra-budgetary resources and technical support from STI, local partners were committed to incorporate RUMA into existing activities at the municipal level. Qualified personnel and opportunities for integration, synergy and co-ordination were identified during the meetings with local partners and the collaborations were always very successful.

The RUMA methodology is a cross-sectional design and the results are likely to change over time due to seasonality, the dynamics of urbanization and the evolution of malaria transmission. In Dar es Salaam, for example, the surveys were carried out during an exceptionally dry period and results could underestimate the true transmission intensity. Many factors such as the size of the city, the fieldwork logistics, the availability of local expertise and the coordination with local senior officers can influence the schedule and planning, as well as the outcome of such surveys.

The study highlighted the need for improved Health Management Information Systems (HMIS) in SSA urban areas. Municipal health departments routinely collect health facility data but information is rarely fed back to the districts and facilities that generate the information. The data are often not available for analysis or accessible due to false registration and under-reporting from health facilities, as well as poor filing and storage of documents at the district or municipal level. In addition, the low number of true malaria cases among fever episodes treated as "malaria" raises the issue of the validity of the collected data even further. Hence, much progress needs to be made in order to estimate more accurately the urban malaria burden and plan relevant control measures.

GIS provides a platform to display health services and geographic features in relation to population settlements. In this experience policy-makers could readily use the presented information for improved planning, re-allocation of resources and for strengthening the networking between the public and private sectors. While the GIS technology has been shown to be very useful in studying health care delivery and distribution of diseases, its application in an entomological assessment was quite difficult and costly and could only be done in conjunction with other ongoing projects. Hence it should be excluded from the process of RUMA. In contrast, the mapping of health facilities with GIS was feasible and cost-effective.

While results from the school surveys gave an indication of the endemicity range and risks in the targeted community, they cannot be considered as being representative without a wider survey. The variations of malaria risk were sometimes related to political divisions or man-made boundaries, but often were due to divergent socio-environmental factors and the degree of urbanization. Because site-specific environmental conditions lead to an aggregated distribution of vectors and different malaria risks, the sampling sites were selected taking into account the population density, the natural environment and urbanization patterns. This should improve the rough categories that previous researchers applied (centre, intermediate and periphery).

Despite a potential attendance bias, the health facility surveys allowed the determination of prevalence of parasitaemia among presenting clinical cases, and the calculation of the fraction of malaria-attributable fevers. This allowed to document clearly the high rate of malaria mis-diagnosis in the health facilities. This information is of great importance for urban malaria control.

Overall, RUMA is a first step towards understanding malaria endemicity and designing control strategies. It has exemplified the concern for mis-diagnosis of clinical malaria in SSA cities [25,50]. A report by the Tanzania-Japan malaria control programme in Dar es Salaam mentioned that drug administration to diagnosed children was one of the essential interventions that reduced the malaria rates between 1988 and 1996 [36]. An in-depth research is now being implemented in Dar es Salaam to assess the malaria burden with a much larger sample size. The application of RUMA methodology is possible and desirable in other SSA urban areas and it should have a special focus on improved diagnosis.

## List of abbreviations

CHU Centre Hospitalier Universitaire

CNRFP Centre National de Recherche et de Formation sur la Paludisme, Burkina Faso

CREC Centre de Recherche Entomologique de Cotonou

CSRS Centre Suisse de Recherches Scientifiques, Côte d'Ivoire

EIER Ecole Inter-Etats d'Ingénieurs et de l'Equipement Rural, Burkina Faso

GIS Geographic Information System

HMIS Health Management Information Systems

ITNs Insecticide-Treated Nets

MOH Ministry of Health

RUMA Rapid Urban Malaria Appraisal

SSA Sub-Saharan Africa

STI Swiss Tropical Institute

## Authors' contributions

SW participated in the design of the study, conducted the field work, analysed and interpreted data and drafted the manuscript. CL conceived the study, coordinated the field work and revised the manuscript. TS and PV assisted in the design and the statistic analysis. CG, DD, MA and DM were the key local contacts, facilitated the collaboration and supervised the data collection and laboratory works at each site. AT participated in the design of the study. MT participated in the conception of the work, facilitated the overall coordination and revised it critically at all stages.

## Supplementary Material

Additional File 1 the questionnaire for health facility-based survey Click here for file

Additional File 2 the questionnaire for school parasitaemia survey Click here for file
