# Supplementary material for: Rapid urban malaria appraisal (RUMA) in sub-Saharan Africa
Source: Malar J. 2005 Sep 9;4:40. doi: 10.1186/1475-2875-4-40 (PMC1249588; doi:10.1186/1475-2875-4-40)
Supplement: Additional File 1 — the questionnaire for health facility-based survey [file 1475-2875-4-40-S1.doc]

# Form 1 Parasitaemia Survey in the Schools (English version)

Swiss Tropical Institute, in collaboration with World Health Organisation and Dar es Salaam City Council, are glad to inform you that our survey team will conduct a malaria survey in the school. Dar es Salaam city council is planning a malaria control program, thus we need to collect the basic information. We will take the blood smear and exam whether your child has malaria parasite. The procedure will be safe and easy. We will provide the free laboratory examination and treat the clinical malaria **without any charge**. Please kindly fill and return the form, thus the doctor can treat your child properly. Our team will arrive in the school this week.

**You AGREE your child to participate in the survey: Yes____or No___ Name of child: _________________**

1. Where do you live: Ward: ___________Street____________________
2. Where the child born: 1) Dar es Salaam 2) other urban area 3) rural area 4) other country __
3. How long the child have been in Dar es Salaam: __________years
4. Have your child been or travelled in a rural area in last 3 months: 1) Yes or 0) No___

4.a Dates: ____/____ Locations: ______________________________ Duration: ________

4.b Dates: ____/____ Locations: ______________________________Duration: _______

1. Have the child ever been sick of malaria last month? 1) Yes or 0) No___, if no, go to 7
2. Where was your child treated for malaria last time: 1) home 2) health centre/dispensary 3) referral hospital 4) traditional healer or herbs 5) let them sleep 6) no treatment 7) others _________
3. Who is responsible for family health at home? 1) Mother 2) father 3) elderest sister or brother 4) grand-parents 5) other family member 6) others___
4. Did your child sleep in the mosquitoes net last night: 1) Yes or 0) Non __
5. Your mosquitoes net were treated by insecticide? 1) Yes or 0) Non __
6. Housing style: 1) concrete/brick 2) Nyumba ya Malcuti 3) Nyumba ya Bati 4) others___
7. Your water resource: 1) Tap water 2) well 3) public fountain/pool 4) water tank 5) river 6) other___
8. How much money you spent on lunch per day? ___________Your income resource: ___________
9. Do your child have allergy or feel uncomfortable with SP? 1) Yes or 0) No___

----------------------------------------------------------------------------------------------------------------------------

1. Axillary Temperature: ___ ___. ___ ^0^C
2. Serial number ___-___-________(1-250)
3. Survey site: ____________ ward:____________
4. Age: _____ years ____ months or ___/___/____(dd/mm/yy)
5. Sex: 1) Male or 2) Female____

# Laboratory result

# Plasmodium 1) Positive or 0) Negative__ Total density __________*µ*L/blood

| Density | *P. falciparum* | *P. Vivax* | *P. Ovale* | *P. malariae* |
| --- | --- | --- | --- | --- |
| Trophozoite |  |  |  |  |
| Scehizoite |  |  |  |  |
