# Supplementary material for: Rapid urban malaria appraisal (RUMA) in sub-Saharan Africa
Source: Malar J. 2005 Sep 9;4:40. doi: 10.1186/1475-2875-4-40 (PMC1249588; doi:10.1186/1475-2875-4-40)
Supplement: Additional File 2 — the questionnaire for school parasitaemia survey [file 1475-2875-4-40-S2.doc]

# Form 2 Passive Case Detection in the Health Facilities (English version)

**Criteria of enrolment of survey**

1. How long have you been in Dar es Salaam: __________years
2. Have you been treated for your illness before this visit 1) No, 2) Home therapy, 3) Go to referral hospital or other health centres, 4) For the control check, 5) Herbal, 6) Give Paracetamol 7) Give chloroquine 8) Traditional healer 9) Go to pharmacy 10) Others___
3. If others, please specify: __________________________
4. Axillary Temperature: ___ ___. ___ ^0^C
5. How many days the fever have been presented before visiting: ___days
6. 1) CASE / 0) CONTROL __
7. Serial number ___-___-________(1-400) and name of patients_______________________
8. Survey site:____________ ward:____________
9. Location of patient’s residence: Ward: ___________Street____________________ (Mark location on map)

1) Centre, 2) Intermediate, 3) Peri-urban, 4) Rural areas___

1. Age: _____ years _____ months or ___/___/____(dd/mm/yy)
2. Sex: 1) Male or 2) Female____

## Social Economic state

1. Education: ________years (care taker or patient if > 5 years):1) Primary school 2)Secondary school 3) College or higher degree 4) non 5) others_____________
2. Mosquitoes net use in last night: 1) Yes or 0) N __
3. Your mosquitoes net treated by insecticide? 1) Yes or 0) N __
4. Housing style: 1) concrete/brick 2) Nyumba ya Malcuti 3) Nyumba ya Bati 4) others___
5. What’s your water resource: 1) Tap water 2) well 3) public fountain or pool 4) water tank 5) river 6) others___
6. Do you have or do you live near by the agriculture land or garden? 1) Yes or 0) N _
7. How much money you spent on lunch per day? ___________Your income resource:___________
8. How much money you are able to spend on preventing mosquitoes biting per month? ________

**Travel and malaria history**

1. Have you been or travelled in a rural area in last 3 months: 1) Yes or 0) No___, if no, go to 21

20.a Dates: ____/____ Locations: ______________________________ Duration: ________

20.b Dates: ____/____ Locations: ______________________________Duration: ________

1. Where is the patient’s birthplace: 1) Dar es Salaam 2) other urban area 3) rural area 4) other country __
2. Have the patient ever been treated for malaria last month? 1) Yes or 0) No___, if no, go to 24
3. Where were you or your child treated for malaria last time: 1) home 2) pharmacy or drug outlet 3) health centre/dispensary 4) referral hospital 5) traditional healer or herbs 6) just go to sleep 7) several of these 8) no treatment 9) others ____________________________________________

**Clinical diagnosis**

1. Do you have allergy or feel uncomfortable with SP? 1) Yes or 0) No___
2. What symptoms do you have? 1) Fever 2) cold 3) diarrhoea 4) vomit 5) headache 6) dizziness 7) stomach ach 8) back pain 9) lack of appetite 10) others______________(multiple choice)
3. Clinical Diagnoses: ______________________________________
4. Doctor’s compliance on prescription of drug dose: ____________________________________

# Laboratory Result

**Plasmodium: 1) Positive or 0) Nagative___Total density ______________*µ*L/blood**

| Density | *P. falciparum* | *P. Vivax* | *P. Ovale* | *P. malariae* |
| --- | --- | --- | --- | --- |
| Trophozoite |  |  |  |  |
| Scehizoite |  |  |  |  |
| Gametocyte |  |  |  |  |
